# Construct validity and reliability of the 2-Minute Step Test (2MST) in individuals with low back pain

**DOI:** 10.1186/s12891-022-06050-w

**Published:** 2022-12-05

**Authors:** Sulamizia Filomena Costa de Jesus, Daniela Bassi-Dibai, André Pontes-Silva, Aliny da Silva de Araujo, Silvana de Freitas Faria Silva, Christiano Eduardo Veneroso, Cid André Fidelis de Paula Gomes, Almir Vieira Dibai-Filho

**Affiliations:** 1 Postgraduate Program in Physical Education, Universidade Federal do Maranhão, São Luís, MA Brazil; 2 Specialized Center for Rehabilitation and Health Promotion, São Luís, MA Brazil; 3 Postgraduate Program in Environment, Universidade Ceuma, São Luís, MA Brazil; 4 Department of Physical Therapy, Faculdade Santa Terezinha, São Luís, MA Brazil; 5 Postgraduate Program in Physical Therapy, Department of Physical Therapy, Universidade Federal de São Carlos, Rod. Washington Luís, Km 235, CEP, São Carlos, SP 13565-905 Brazil; 6 Postgraduate Program in Rehabilitation Sciences, Universidade Nove de Julho, São Paulo, SP Brazil

**Keywords:** Chronic pain, Musculoskeletal disorders, Reproducibility of results, Cardiorespiratory Fitness

## Abstract

**Background:**

Functional tests are important clinical tools, since they are non-invasive methods, with simple applicability, and low cost. However, there are few tests adapted for individuals with chronic low back pain. Thus, our objective was to evaluate the test-retest and inter-rater reliability of the 2-Minute Step Test in individuals with chronic low back pain and to correlate the test score with measures of pain and physical activity.

**Methods:**

We included patients aged between 18 and 45 years, of both sexes, and with low back pain. The interval between the test and retest was 7 days. We analyzed the data via intra-class correlation coefficient (ICC), confidence interval at 95%, standard error of measurement, and minimum detectable change for reliability. We used the Spearman’s correlation coefficient to verify the correlation between 2-Minute Step Test and measures of pain and physical activity (Numerical Pain Scale, Roland-Morris Disability Questionnaire, Pain-Related Catastrophizing Thoughts Scale, Tampa Scale of Kinesiophobia, and Baecke Habitual Physical Activity Questionnaire).

**Results:**

Sample is composed of 37 individuals, most of them female, with overweight and low back pain > 60 months. 2-Minute Step Test showed excellent test-retest (ICC = 0.903) and inter-rater (ICC = 0.925) reliability. Sport domain of the Baecke showed a significant correlation with the 2-Minute Step Test (rho = 0.444).

**Conclusion:**

2-Minute Step Test is a reliable measure to measure the functional capacity of patients with chronic low back pain considering different times and examiners, as well as being positively correlated with sports practice.

## Introduction

Chronic low back pain is a musculoskeletal disorder that generates disability in affected individuals. To understand the clinical aspects, it is necessary to assess pain intensity, quality of life, and disability [1]. Disability assessment shows limitations individuals with chronic low back pain face in daily living. For this, the most used questionnaires are: Roland-Morris Disability Questionnaire and the Oswestry Disability Index [2]. Besides questionnaires (whose assessment occurs via self-report of the patients), functional tests are important clinical tools, as they are non-invasive methods, with simple applicability, and low cost; however, there are few tests adapted for individuals with chronic low back pain [3, 4].

The 6-Minute Walk Test (6MWT) [4, 5] and the 10-Meter Walk Test (10MWT) [6] are relevant clinical options, but in its applicability the examiner will need ample space or a treadmill (and this reduces the accessibility of the test) [4, 5]. In addition, these tests limited to assess cardiorespiratory fitness [6]. Another option is the 2-Minute Step Test (2MST) used to assess aerobic and functional capacity without using equipment [7–9]. This test is measured a maximum number of knee elevations (steps) that the individual can perform in 2 minutes (during a stationary gait, without displacing the body). The examiner checks the number of steps performed on the right knee, at an intermediate height between the patella and the anterior superior iliac spine [10].

Currently, the 2MST occupies an important position among many tests used to monitor functional status and establish prognosis in individuals with systolic heart failure [11], elderly [12], obese [13], and individuals with knee osteoarthritis [7]. However, we emphasize that, in the same way as instruments used in the clinical environment and in scientific research (questionnaires, forms, and scales), the 2MST should also be investigated regarding the reliability and validity of the measure for low back pain, because it is a test that evaluates sensitive variables such as spatio-temporal parameters of gait which, in turn, may be altered in individuals with chronic low back pain [14].

Some studies have verified the reliability of the 2MST in different populations, via intraclass correlation coefficient (ICC), e.g., knee osteoarthritis (ICC ≥ 0,94) [7], active and sedentary adults (ICC ≥ 0,83) [8]; however, reliability of the 2MST has not been investigated in patients with chronic low back pain. The absence of this investigation makes it impossible to use this test in individuals with low back pain, also impairing evidence-based clinical practice.

Thus, under the hypothesis that the 2MST is a reliable instrument to assess functional capacity in individuals with chronic low back pain (at different assessment times and by different examiners) and is adequately correlated with habitual physical activity, our aim was to evaluate the test-retest and inter-rater reliability of 2MST in subjects with chronic low back pain; and to correlate the test score with measures of pain and physical activity.

## Methods

### Study design

This reliability study was based on Guidelines for Reporting Reliability and Agreement Studies [15] and carried out in specialized rehabilitation centers located in the cities of São Luís and Buriticupu (Maranhão, Northeastern Brazil). The study procedures were approved by the Research Ethics Committee of the institution (protocol 2.965.566). All methods were carried out in accordance with relevant guidelines and regulations. All volunteers included in the study validated their participation by signing a free and informed consent form. Recruitment of volunteers took place at the research site, through verbal dissemination, and on the Internet.

The evaluation procedures were performed by three researchers. A researcher with previous experience with the survey instruments conducted an initial interview with each of the volunteers, applied the eligibility criteria, and the instruments: Numeric Pain Rating Scale (NPRS), Roland-Morris Disability Questionnaire (RMDQ), Pain-Related Catastrophizing Thoughts Scale (PCTS), Tampa Scale of Kinesiophobia (TSK), Baecke Habitual Physical Activity Questionnaire (BQ). Then, two other examiners previously trained and familiarized with the 2MST performed the functional capacity assessments in two moments (using an interval of 1 week) [16].

### Sample

We used an online calculator for sample calculation, available at the website: https://wnarifin.github.io/ssc/ssicc.html. The parameters used for the calculation were: expected ICC value = 0.83, minimum acceptable ICC value = 0.60, significance level of 5% and statistical power of 80% [8]. Thus, the resulting sample size was 37 participants.

The sample consisted of individuals with chronic low back pain, both sexes, aged from 18 to 45 years. We considered the following non-inclusion criteria: individuals with lumbar disc herniation; history of fracture in the lumbar; surgery in the spine or lower limbs; presence of pain in any region of the lower limbs; medical diagnosis of fibromyalgia, labyrinthitis, cognitive dysfunction, or any disease causes balance alteration [17].

### Numeric Pain Rating Scale (NPRS)

NPRS was validated for Portuguese by Ferreira-Valente et al. [18]. It is a subjective scale with intervals from 0 to 10, in which 0 represents “no pain” and 10 represents “worst pain imaginable”. We measured pain intensity at rest and after active lumbar spine movements.

### Roland-Morris Disability Questionnaire (RMDQ)

RMDQ was validated for the Brazilian population by Nusbaum et al. [19]. It is a valid and reliable measure for Brazilians with low back pain. It is a questionnaire composed of 24 items with two response options: no (which is equivalent to a value of 0) and yes (which is equivalent to a value of 1). Total score ranges from 0 (suggesting no disability) to 24 (severe disability).

### Tampa Scale of Kinesiophobia (TSK)

TSK was validated for the Brazilian population by Siqueira et al .[20], composed of 17 questions related to fear of movement in the presence of pain. For each item, the TSK presents the following response options: totally disagree (1 point), partially disagree (2 points), partially agree (3 points), and totally agree (4 points). For the final calculation, it is necessary to invert the scores of questions 4, 8, 12, and 16. The minimum score is 17 and the maximum is 68; higher the score, the greater the degree of kinesiophobia.

### Pain-Related Catastrophizing Thoughts Scale (PCTS)

PCTS was validated for the Brazilian population by Sardá-Junior et al. [21] to measure the catastrophizing related to chronic pain. This scale is composed of 9 items with 5 response options (ranging from “almost never” to “almost always”). Total score is the sum of the items divided by the number of items answered, with the minimum score being 0 and the maximum 5; higher values indicate greater catastrophizing.

### Baecke Habitual Physical Activity Questionnaire (BQ)

BQ was validated for the Brazilian population by Florindo and Latorre [22], composed of 16 questions related to habitual physical activity over a twelve-month interval. It has 3 domains: occupational, sport, and leisure. Score for each domain ranges from 1 to 5; higher scores indicate greater habitual physical activity.

### 2-Minute Step Test (2MST)

2MST is a test to assess functional capacity. We counted the maximum number of knee elevations that the individual can perform in 2 minutes, in which the minimum height occurs at a midpoint of the distance between the patella and the anterior superior iliac spine (during a stationary gait, without displacing the body) [12]. We counted the maximum number of knee elevations that the individual can perform in 2 minutes, in which the minimum height occurs at a midpoint of the distance between the patella and the anterosuperior iliac spine [11]. We apply the test in a temperature-controlled room (~ 23 °C). At the examiner’s indicative signal, the participant was encouraged to start a stationary gait. Examiner recorded the maximum number of elevations (steps) that the volunteer performed with the right knee for 2 minutes. Then the volunteer rested for 5 minutes and the second evaluator repeated the test (inter-rater reliability). After 7 days, the volunteers repeated this entire procedure (test-retest reliability).

### Statistical analysis

We used the ICC_2,3_ to determine test-retest and inter-rater reliability in measuring the functional performance of individuals, with their respective confidence interval at 95%, standard error of measurement (SEM), and minimum detectable change (MDC). The interpretation of the ICC value was based on the study by Fleiss [23]: < 0.40 = low reliability; from 0.40 to 0.74 = moderate reliability; from 0.75 to 0.90 = substantial reliability; > 0.90 = excellent reliability. We use the formulas: SEM = standard deviation × √(1 – ICC) and MDC = 1.96 × SEM × √2 [24].

Furthermore, after identifying the non-normality of the data distribution using the Shapiro-Wilk test, we used the Spearman’s correlation coefficient (rho) to verify the magnitude of the correlation between the 2MST and measures of pain and physical activity. We used the classification established by Zou et al. [25] to interpret the magnitude of correlations: 0 = no correlation; 0.20 = weak correlation; 0.50 = moderate correlation; 0.80 = strong correlation; 1.00 = perfect correlation. We processed these data in SPSS software, version 17.0 (Chicago, IL, USA), with a significance level of 5% in all analyses.

## Results

A total of 41 subjects were recruited for the study; there was a sample loss of 4 participants not attending the retest. Thus, the final sample consisted of 37 participants, most composed of women, adults, overweight, and with low back pain > 60 months (Table 1). Table 2 presents the 2MST values.

**Table 1:** Sample characteristics (*n =* 37)

Variables	Values
Age (years), mean (SD)	32.48 (9.06)
Sex (female), n (%)	31 (83.8)
Education, n (%)	
Basic	27 (73)
Higher	10 (27)
Smoking (no), n (%)	37 (100)
Alcoholism (no), n (%)	25 (67.6)
BQ (score), mean (SD)	
Occupational	2.77 (0.48)
Sport	2.13 (0.73)
Recreation	2.14 (0.61)
Body mass (kg), mean (SD)	67.36 (18.18)
Stature (m), mean (SD)	1.61 (0.07)
Body mass index (kg/m^2^), mean (SD)	25.70 (6.18)
Pain duration (months), mean (SD)	64.94 (51.40)
NPRS (score), mean (SD)	
At rest	5.24 (2.36)
After movements	5.83 (2.14)
PCTS (score), mean (SD)	2.19 (1.18)
TSK (score), mean (SD)	42.89 (8.48)
RMDQ (score), mean (SD)	8.16 (5.05)

*BQ* Baecke Habitual Physical Activity Questionnaire, *NPRS* Numeric Pain Rating Scale, *PCTS* Pain-Related Catastrophizing Thoughts Scale, *TSK* Tampa Scale of Kinesiophobia, *RMDQ* Roland-Morris Disability Questionnaire, *SD* Standard deviation

**Table 2:** Mean (standard-deviation) of the 2-Minute Step Test (2MST) in individuals with low back pain according to the 2 examiners (*n =* 37)

Examiner 1		Examiner 2	
Test	Retest	Test	Retest
55.78 (16.65)	58.56 (18.99)	53.18 (16.14)	53.48 (17.94)

We identified excellent test-retest and inter-rater reliability (ICC > 0.90; SEM < 10%; MDC ≥ 25%), as shown in Tables 3 and 4, with ICC values above 0.75 (acceptability cut-off point).

**Table 3:** Test-retest reliability of the measurement of the 2-Minute Step Test (2MST) in individuals with low back pain (*n =* 37)

Variable	ICC	95% CI	SEM	SEM (%)	MDC	MDC (%)
2MST	0.903	0.811, 0.950	5.31	9.95	14.71	27.58

*ICC* Intraclass correlation coefficient, *CI* Confidence interval, *SEM* Standard error of measurement, *MDC* Minimum detectable change

**Table 4:** Inter-rater reliability of the measurement of the 2-Minute Step Test (2MST) in individuals with low back pain (*n =* 37)

Variable	ICC	95% CI	SEM	SEM (%)	MDC	MDC (%)
2MST	0.925	0.855, 0.961	5.06	9.03	14.02	25.02

*ICC* Intraclass correlation coefficient, *CI* Confidence interval, *SEM* Standard error of measurement, *MDC* Minimum detectable change

**Fig. 1:**
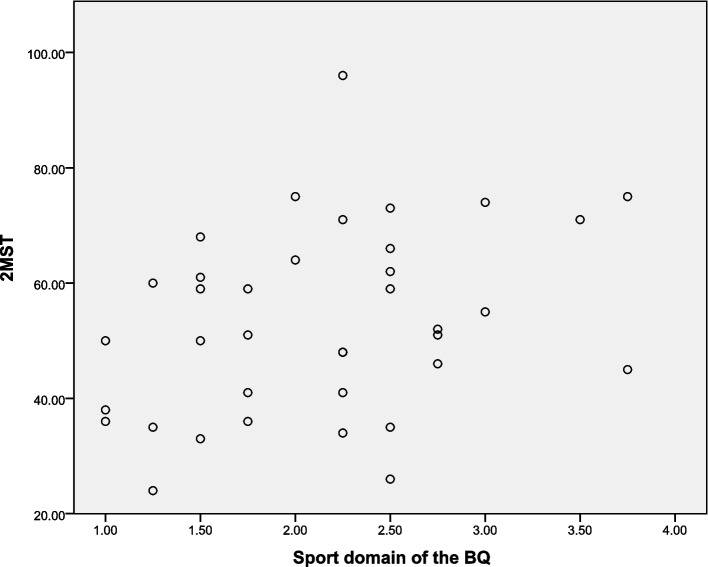
Correlation between the 2-Minute Step Test (2MST) and the sport domain of the Baecke Habitual Physical Activity Questionnaire (BQ)

Additionally, when the correlation between the 2MST and the pain and physical activity variables was performed, we identified a weak and significant correlation (rho = 0.444, *p <* 0.05) with the sport domain of the BQ (Table 5 and Fig. 1). However, no significant correlations were observed between the measures of pain and the occupational and leisure domains of the BQ (*p >* 0.05).

**Table 5:** Correlation between 2-Minute Step Test (2MST) and other tools (*n =* 37)

Variables	2MST
BQ	
Occupational	rho = 0.147, *p =* 0.384
Sport	rho = 0.444, *p =* 0.006 *
Recreation	rho = 0.219, *p =* 0.193
NPRS	
At rest	rho = −0.296, *p =* 0.075
After movements	rho = − 0.077, *p* = 0.651
PCTS	rho = − 0.177, *p =* 0.295
TSK	rho = − 0.315, *p =* 0.058
RMDQ	rho = − 0.269, *p =* 0.107

*BQ* Baecke Habitual Physical Activity Questionnaire, *NPRS* Numeric Pain Rating Scale, *PCTS* Pain-Related Catastrophizing Thoughts Scale, *TSK* Tampa Scale of Kinesiophobia, *RMDQ* Roland-Morris Disability Questionnaire; rho: Spearman’s correlation coefficient

* Significant correlation (*p <* 0.05)

## Discussion

The literature is scarce regarding the reliability of this test in the most diverse populations. However, like our results, a few existing studies also found adequate reliability. Rikli and Jones [26] identified excellent reliability of the 2MST in independent elderly (ICC value = 0.90). Nogueira et al. [8] identified adequate test-retest and inter-rater reliability of 2MST in active and sedentary adults (ICC values ≥0.83); in addition, corroborating our results, previous study observed a significant, positive, and weak correlation between the 2MST and the occupational domain of the BQ [8].

When verifying the reliability of 2MST in individuals with symptomatic peripheral arterial disease, Braghieri et al. [27] point out that the test is reliable (ICC = 0.945), significantly correlates with the 6-minute walk test (rho = 0.55), and promotes lower cardiovascular stress. De Morais Almeida et al. [7] also found similar results to our study: excellent reliability in test-retest and inter-rater (ICC = 0.94 and 0.97, respectively) of 2MST in individuals with knee osteoarthritis, as well as adequate correlation with self-efficacy (rho = 0.503), disability (rho = 0.536), pain intensity at rest (rho = 0.347), and pain intensity on movement (rho = 0.478).

BQ is constantly used to assess habitual physical activity in the most diverse populations [28–31]. BQ assesses the occupational, sport, and recreation domains [32]. In our study, we found a significant correlation between the 2MST and the sport domain of BQ, these findings may indicate that sport is recommended to maintain good levels of cardiorespiratory fitness in people with chronic low back pain. Patients with chronic low back pain are vulnerable populations, as they have lower levels of strength [33], aerobic capacity [34], VO2max [35], and metabolic thresholds, negatively impacting the walking economy, quality of life, and practice of physical exercise [6]. The 2MST is a strategy to assess these patients without exposing them to the maximum physical exertion tests, respecting their biological individuality as well as their disability.

It is important to highlight that all tests submitted for reliability evaluation must present the SEM and MDC. In conceptual terms, SEM is a measure that reflects the error inherent in an evaluative test [36], and for 2MST we observed an error of less than 10%. There is no consensus in the literature for the best cut-off point for SEM, however, a previous study establishes 10% as the acceptability cut-off point [36].. In previous studies of 2MST in other populations, SEM ≤ 7.50 and ≤ 6.72 were observed in the study conducted by Nogueira et al. [8] with healthy individuals and in the study conducted by De Morais Almeida et al. [7] with patients with knee osteoarthritis, respectively. Braghieri et al. [27] did not present SEM in a study of patients with peripheral arterial disease.

In turn, MDC is the minimum value that must be considered to state that a change in a score or a test result is error free [37]. The MDC is related to the SEM value and, to the best of our knowledge, there is no defined cut-off point in the scientific literature. Our study observed MDC values of 14.71 steps (27.58%) and 14.01 steps (25.02%) for test-retest and inter-examiner reliability, respectively. The MDC values in the studies conducted by Nogueira et al. [8], De Morais Almeida et al. [7], and Braghieri et al. [27] were ≤ 24.10 steps, ≤ 12.40 steps, and 3.2 steps respectively.

A systematic review by Bohannon and Crouch [38] evaluated the clinimetric properties and supported the use of 2MST for some diseases (e.g., heart failure, osteoporosis, Parkinson’s disease, and so on), but the chronic low back pain was not among the diseases researched (our study was the first to assess the reliability of the 2MST in individuals with chronic low back pain). We suggest that researchers conduct further studies to address the validity, reliability, and responsiveness of 2MST in other diseases.

2MST has proven to be a reliable test for many populations, including chronic low back pain. Our study supports the use of this test in small clinical spaces, therefore, we overcome this limitation mentioned by Carvalho et al. [6]. However, this study has limitations that should be highlighted. Correlations performed with the 2MST were based on self-report instruments (scales and questionnaires) and there was no correlation with another test that measures functional capacity (e.g., Sit-to-Stand Test, 6-Minute Walk Test, 10-Meter Walk Test, and so on), as there is a scarcity of validated functional tests for patients with low back pain. Besides, the sample was predominantly female and there was no analysis of the reliability of the 2MST according to sex. In addition, it was not possible to determine categories or cut-off points to provide clinical support, therefore, we suggest further studies based on these gaps.

## Conclusion

2MST is a reliable measure to measure the functional capacity of patients with chronic low back pain considering different times and examiners, as well as being positively correlated with sports practice. Finally, we suggest that this test be used in clinical practice and in research with individuals with chronic low back pain.

## Data Availability

The datasets used and/or analysed during the current study available from the corresponding author on reasonable request.
